# The association between drospirenone and hyperkalemia: a comparative-safety study

**DOI:** 10.1186/1472-6904-11-23

**Published:** 2011-12-30

**Authors:** Steven T Bird, Salvatore R Pepe, Mahyar Etminan, Xinyue Liu, James M Brophy, Joseph AC Delaney

**Affiliations:** 1 Department of Health and Human Services/Food and Drug Administration/Center for Drug Evaluation and Research (CDER)/Office of Management/CDER Academic Collaboration Program, Bldg 22, 10903 New Hampshire Avenue, Silver Spring, MD USA 20993; 2 University of Florida, College of Pharmacy, Pharmaceutical Outcomes & Policy, 101 S. Newell Drive (HPNP), PO Box 100496, Gainesville FL, USA 32611; 3 University of British Columbia, Pharmaceutical Outcomes Programme, 709-828 West 10th Avenue, Vancouver, British Columbia, Canada V5Z1M9; 4 McGill University, Royal Victoria Hospital, 687 Pine Street West, Montreal, Quebec H3A 1A1, Canada

## Abstract

**Background:**

Drospirenone/ethinyl-estradiol is an oral contraceptive (OC) that possesses unique antimineralocorticoid activity. It is conjectured that drospirenone, taken alone or concomitantly with spironolactone, may be associated with an increased risk of hyperkalemia.

**Methods:**

A retrospective cohort study was conducted evaluating women between 18-46 years of age in the Lifelink™ Health Plan Claims Database. The study was restricted to new users of OCs between 1997-2009. Cox proportional hazards models were used to estimate the time to first occurrence of hyperkalemia diagnosis. The main analysis compared OCs containing drospirenone with OCs containing levonorgestrel, a second generation OC not known to impact potassium homeostasis. Logistic regression evaluated concomitant prescribing of drospirenone and spironolactone

**Results:**

The cohort included 1,148,183 women, averaging 28.8 years of age and 280 days of OC therapy. 2325 cases of hyperkalemia were identified. The adjusted hazard ratio (HR) for hyperkalemia with drospirenone compared to levonorgestrel was 1.10 (95%CI 0.95-1.26). There was an increased risk of hyperkalemia with norethindrone HR 1.15 (95%CI: 1.00-1.33) and norgestimate HR 1.27 (95%CI: 1.11-1.46). Other OCs were unassociated with hyperkalemia. The odds of receiving spironolactone while taking drospirenone were 2.66 (95%CI 2.53-2.80) times higher than the odds of receiving spironolactone and levonorgestrel. Only 6.5% of patients taking drospirenone and spironolactone had a serum potassium assay within 180 days of starting concomitant therapy.

**Conclusions:**

A clinically significant signal for hyperkalemia with drospirenone was not demonstrated in the current study. Despite the bolded warning for hyperkalemia with joint drospirenone and spironolactone administration, physicians are actually using them together preferentially, and are not following the recommended potassium monitoring requirements in the package insert.

## Background

Drospirenone is a novel synthetic progestin approved in combination with ethinyl estradiol as an oral contraceptive (OC) [1]. Marketed as Yasmin^® ^and Yaz^®^, drospirenone is one of the most popular oral contraceptives in the United States [2]. Drospirenone is a fourth generation OC and it possesses antimineralocorticoid effects not present in previous generations of OCs. Its antimineralocorticoid potency is approximately eight times greater than spironolactone [3], thus a 3 mg tablet of drospirenone has a similar effect to 20-25 mg of spironolactone [4]. This activity enhances sodium, chloride, and water excretion, while reducing the excretion of potassium, ammonium, and phosphate [5]. The similarity in chemical structure between drospirenone and spironolactone and the known association between spironolactone and hyperkalemia both strengthen the plausibility that clinically significant hyperkalemia might result from drospirenone use.

In May of 2001, when drospirenone/ethinyl estradiol (Yasmin^®^) was first approved, the package insert included a bolded warning for hyperkalemia, stating that "Yasmin should not be used in patients with conditions that predispose to hyperkalemia" [1]. The warning also instructs physicians to monitor potassium levels during the first cycle of treatment in patients taking concomitant medications known to cause hyperkalemia. Clinical evidence however has not shown a strong association between drospirenone and hyperkalemia [6-13]. Several studies have evaluated for hyperkalemia in postmenopausal women with hypertension or diabetes who use drospirenone to treat vasomotor spasms. These studies found no association between drospirenone and hyperkalemia in women with hypertension [6,7] or type 2 diabetes mellitus [8]; however, all three studies were twelve or fewer weeks in duration. Larger trials designed to evaluate the safety and efficacy of drospirenone either do not evaluate hyperkalemia or are not powered to detect it [9-12]. Only one large study, mandated by the FDA at approval, has been performed evaluating drospirenone for hyperkalemia in younger women, and it found no association between drospirenone and hyperkalemia [13].

Hyperkalemia is a potentially serious condition that may be associated with numerous pathophysiological conditions. Clinically significant hyperkalemia reduces membrane excitability and disturbs the acid-base balance, manifesting as weakness, flaccid paralysis, hypoventilation, and metabolic acidosis. Hyperkalemia can also result in cardiac toxicity with electrocardiographic changes, which in severe cases may lead to the terminal events of ventricular fibrillation or asystole [5].

The primary objective of the current study is to investigate the association between drospirenone and the diagnosis of hyperkalemia in a large unselected population. A secondary objective is to evaluate the impact of the package insert on medical prescribing as assessed by an examination of the 1) concomitant use of drospirenone and spironolactone and 2) respect for the stated potassium monitoring requirements.

## Methods

### Data Source

The IMS Lifelink™ Health Plan Claims Database contains paid claims data from over 102 managed care plans in the United States. The database contains fully adjudicated medical and pharmacy claims for over 68 million patients, including inpatient and outpatient diagnoses and procedures (*International Classification of Diseases, 9^th ^Revision, Clinical Modification format*) in addition to retail and mail order prescription records. The data is representative of US residents with private health insurance in terms of geography, age, and gender. The Lifelink™ database is subject to quality checks to ensure data quality and to minimize error rates [14].

### Cohort description

A retrospective cohort was developed, evaluating women in the Lifelink™ Claims database between January 1^st^, 1997 and December 31^st^, 2009. All women between 18-46 years of age with the first prescription for an OC containing ethinyl estradiol (0.35 ug or less) and one of the following progestins were included in the cohort: *desogestrel, drospirenone, ethynodiol diacetate, levonorgestrel, norethindrone acetate, norethindrone, norgestimate, and norgestrel*. All patients who met these inclusion criteria were analyzed in the utilization portion of the study.

For the hyperkalemia analysis, in order to include only new users, patients were excluded if they did not have at least 180 days of enrollment history prior to their first claim for an OC. Patients were also excluded if they had a prior diagnosis of hyperkalemia. Censoring was performed if a patient switched to another OC during the study period, on the final day of OC possession (determined from the final prescription date and day supply), before a gap in OC possession of 30 or more days, at the event of hyperkalemia, and at the end of the study period, December 31^st^, 2009. Evaluation of hyperkalemia was performed using a diagnostic ICD-9 code (276.7).

### Statistical analysis

#### OCs and hyperkalemia

Cox proportional hazard models were used to estimate the time to first occurrence of hyperkalemia. The primary analysis used a new user design and compared OCs containing drospirenone with OCs containing levonorgestrel. Levonorgestrel was chosen *a priori *as a reference based on its high utilization, lack of association with hyperkalemia, and use as a reference in previous OC comparative-safety studies [15-21]. All estimates were adjusted by age, calendar time, chronic kidney disease, diabetes mellitus, hypertension, inflammatory bowel disease, obesity [22], polycystic ovary syndrome, premenstrual tension syndrome (premenstrual syndrome and premenstrual dysphoric disorder), smoking status, and concomitant medications known to cause hyperkalemia. The following medications were adjusted for: angiotensin-converting enzyme inhibitors (ACE)/angiotensin receptor blockers (ARB), non-steroidal anti-inflammatory drugs (NSAID), spironolactone, and other medications (cyclosporine, diuretics, heparin, penicillin G, tacrolimus, and trimethoprim).

#### Utilization

Concomitant utilization of drospirenone and spironolactone was analyzed during the entire study period. Logistic regression was used to form odds ratios (OR) comparing the odds for receiving concomitant spironolactone and drospirenone therapy against the odds of receiving concomitant spironolactone and levonorgestrel therapy. ORs were also formed calculating the odds of receiving spironolactone while on other progestin-containing OCs compared to levonorgestrel. To evaluate compliance with potassium monitoring for patients taking concomitant drospirenone and spironolactone, the percentage of patients who had a blood serum potassium assay (CPT-4 84132) during the first 180 days of concomitant therapy was calculated.

This study was approved by the University of Florida IRB. All calculations were performed in SAS software version 9.2.

## Results

### OCs and hyperkalemia

The cohort included 1,148,183 women exposed to a progestin-based OC and 880,014 person-years of follow-up time. Patients in the study averaged 28.8 years of age and had a mean follow up time of 280 days. There were 2325 cases of hyperkalemia, representing 0.20% of the population. Baseline characteristics are shown in Table 1.

**Table 1:** Characteristics of women included in the study cohort by type of progestin oral contraceptive used (n = 1,148,183)

	*Desogestrel*	*Drospirenone*	Ethynodiol Diacetate	*Levonorgestrel*	*Norethindrone Acetate*	*Norethindrone *	*Norgestimate*	*Norgestrel*
Number of patients	139,871	224,408	17,295	180,720	93,818	234,105	228,276	29,690
Age	28.7	29.0	28.8	29.0	30.5	29.7	26.9	29.7
Mean follow up (days)	327	272	327	304	240	230	307	249
Number of cases	267	488	33	349	200	433	499	56
**Covariates (%)**								
CKD †	0.15	0.09	0.08	0.09	0.12	0.15	0.08	0.19
Diabetes	4.10	4.10	4.41	3.93	4.13	4.12	3.37	5.43
Hypertension	8.34	8.47	8.53	8.62	9.78	8.89	6.55	10.82
IBD*	0.94	1.03	1.22	1.00	1.03	0.85	0.75	0.98
Obesity	11.59	12.53	12.44	11.22	10.75	10.08	9.50	13.60
PCOS ᶋ	4.47	5.78	5.00	2.11	2.80	2.81	2.21	3.48
PTS (PMS/PMDD) ‡	3.63	5.53	3.04	3.01	3.26	1.95	1.69	2.73
Smoking	6.62	5.94	8.48	7.36	6.43	6.49	6.97	9.00
ACEI/ARB §	0.50	0.47	0.56	0.60	0.75	0.67	0.42	0.80
NSAIDS ʗ	5.67	4.84	5.79	5.56	5.13	5.90	4.77	6.09
Spironolactone	0.43	0.74	0.84	0.28	0.32	0.19	0.32	0.29
Other Medications ʒ	0.46	0.36	0.56	0.46	0.37	0.35	0.27	0.44

*Inflammatory bowel disease

† CKD = Chronic Kidney Disease

ᶋ PCOS = Polycystic Ovary Syndrome

‡ PTS (PMS/PMDD) = premenstrual tension syndrome (premenstrual syndrome and premenstrual dysphoric disorder)

§ ACE/ARB = angiotensin-converting enzyme inhibitors/angiotensin receptor blockers

ʗ NSAID = non-steroidal anti-inflammatory drugs

ʒ Other medications = diuretics, heparin, cyclosporine, tacrolimus, trimethoprim, and penicillin G

The adjusted hazard ratio (HR) for a recorded diagnosis of hyperkalemia while exposed to drospirenone compared to levonorgestrel was 1.10 (95% CI 0.95-1.26). Other OCs were unassociated with hyperkalemia: desogestrel HR 1.00 (95%CI: 0.85-1.17), ethynodiol diacetate HR 0.71 (95%CI: 0.49-1.02), norethindrone acetate HR 1.08 (95%CI: 0.91-1.29), norgestrel HR 1.00 (95%CI: 0.76-1.33), although there was an unexpected signal with norethindrone HR 1.15 (95%CI: 1.00-1.33) and norgestimate HR 1.27 (95%CI: 1.11-1.46) (Table 2). Additionally, the analysis found no interaction between drospirenone and spironolactone for hyperkalemia in the regression model (HR 1.08, 95%CI: 0.78-1.49). Other interactions with drospirenone in the regression model were as follows: ACEI/ARB HR 0.78 (95%CI 0.55-1.10) and NSAID HR 1.09 (95%CI 0.80-1.48).

**Table 2:** Risk for hyperkalemia* with use of commonly used oral contraceptives

	Crude HR (95% CI)	Adjusted HR† (95%CI)
Levonorgestrel	1.0 (reference)	1.0
Desogestrel	0.93 (0.79-1.09)	1.00 (0.85-1.17)
Drospirenone	1.26 (1.10-1.44)	1.10 (0.95-1.26)
Ethynodiol diacetate	0.96 (0.67-1.38)	0.71 (0.49-1.02)
Norethindrone acetate	1.41 (1.18-1.68)	1.08 (0.91-1.29)
Norethindrone	1.27 (1.11-1.47)	1.15 (1.00-1.33)
Norgestimate	1.13 (0.98-1.29)	1.27 (1.11-1.46)
Norgestrel	1.13 (0.85-1.49)	1.00 (0.76-1.33)

*Hyperkalemia determined from a diagnostic ICD-9 code (276.7)

† Adjusted by age, calendar time, chronic kidney disease, diabetes mellitus, hypertension, inflammatory bowel disease, obesity, polycystic ovary syndrome, premenstrual tension syndrome (premenstrual syndrome and premenstrual dysphoric disorder), smoking status, angiotensin-converting enzyme inhibitors/angiotensin receptor blockers, non-steroidal anti-inflammatory drugs, spironolactone, and other medications known to cause hyperkalemia (cyclosporine, diuretics, heparin, penicillin G, tacrolimus, and trimethoprim).

### Utilization

The utilization study evaluated all 2,925,407 patients that met the initial study inclusion criteria. 18,869 patients in this population were taking both spironolactone and an OC. The odds of receiving spironolactone while on drospirenone were 2.66 (95%CI 2.53-2.80) times higher than the odds of receiving spironolactone while on levonorgestrel. The ORs for receiving spironolactone while on other progestin-based OCs compared to levonorgestrel are as follows: desogestrel 1.46 (95%CI 1.38-1.55), ethynodiol diacetate 2.85 (95%CI 2.62-3.11), norethindrone acetate 1.01 (95%CI 0.93-1.08), norethindrone 0.78 (95%CI 0.74-0.83), norgestimate 1.34 (95%CI 1.27-1.41), and norgestrel 0.98 (95%CI 0.89-1.08). The Yasmin^® ^and Yaz^® ^package inserts recommend potassium monitoring within the first treatment cycle for patient taking other medications known to cause hyperkalemia. Of the 5,752 patients who took drospirenone and spironolactone concomitantly, only 376 (6.5%) patients underwent a serum potassium assay within 180 days of starting concomitant therapy.

## Discussion

The current study did not find a substantial and meaningful association between drospirenone use and hyperkalemia compared to patients taking levonorgestrel. It is interesting to note that norethindrone and norgestimate are both associated with a higher risk for hyperkalemia compared to levonorgestrel. These however were not *a priori *hypotheses and may be chance findings due to multiple testing. These results must also be taken into context with the low absolute risk for hyperkalemia in OC users. The increased HR for norethindrone of 1.16 results in a number need to harm (NNH) of 3086 patients, while the HR for norgestimate of 1.27 results in a NNH of 1829 patients.

The null association between drospirenone and hyperkalemia is concordant with the results from previous studies [13,23]. To our knowledge, only one prior cohort study has been conducted with the primary aim to evaluate drospirenone and hyperkalemia [13]. This study had 67,287 OC users, identified 378 cases of hyperkalemia, and found a RR comparing drospirenone to other OCs of 0.9 (95%CI 0.7-1.1). Our study population has approximately seventeen times the OC user population of this prior analysis, allowing greater detection for hyperkalemia and providing increased statistical precision. Another study identified 102 cases of drug-associated hyperkalemia and did not attribute any cases to use of drospirenone [23]. In our study, the comparison among OC users in our analysis minimizes the risk of confounding by indication, and the new user design eliminates the survivor effect that long term OC users are tolerant to the therapy and healthier than short term users. The totality of the evidence suggests that hyperkalemia while on drospirenone is not of clinical importance.

### Utilization

We found that drospirenone users are 2.66 times more likely to receive spironolactone compared with levonorgestrel users. An OR of this magnitude suggests that physicians are not avoiding the concomitant use of drospirenone and spironolactone, but prescribing them together. This is a particularly interesting finding because drospirenone is the only OC with a bolded warning for hyperkalemia. These medications have no overlap in labeled indications; however, drospirenone does have an indication for acne vulgaris, while spironolactone has an off-label use for its treatment. Another likely explanation in the recent literature is that drospirenone and spironolactone are both seen as beneficial for treatment of weight gain and bloating experienced by patients with postmenstrual dysphoric disorder and in reducing hirsutism and acne in patients with polycystic ovarian syndrome (PCOS) [24-28].

It was recently reported that, among 11,019 drospirenone users, 17.6% of patients are taking another medication known to induce hyperkalemia [29]. In this study, spironolactone accounted for 11.1% of this concomitant utilization. Our study found that only 6.5% of patients taking drospirenone and spironolactone underwent potassium monitoring. This raises concern that few physicians are following the recommendations for monitoring serum potassium as stated in the package insert.

Although we found a non-significant interaction for hyperkalemia with concomitant use of drospirenone and spironolactone, this does not assure the safe combined use of these two medications. Particularly, patients with PCOS generally express characteristics of metabolic syndrome, are at an increased risk for drug induced liver injury [30], and warrant careful monitoring.

### Limitations

The use of ICD-9-CM codes for the detection of hyperkalemia provides a high specificity for diagnosed cases because this diagnosis is made from an assay of serum potassium. This measurement however lacks sensitivity due to a lack of potassium testing in the general population. Inadequacies in documenting ICD-9-CM codes could also lead to underreporting of hyperkalemia. To determine if this was likely to be problematic, we interrogated the Lifelink™ database to investigate control drugs with known associations to hyperkalemia. Amiloride, a potassium-sparing diuretic, spironolactone, an aldosterone antagonist, and all ACE inhibitors were selected as positive controls. The Lifelink™ database was able to replicate three known positive associations: amiloride HR 7.94 (95%CI 1.96-32.08), spironolactone HR 3.46 (95%CI 2.97-4.02), and ACE inhibitors HR 1.90 (95%CI 1.70-2.11). Negative controls selected were loratadine, a non-drowsy antihistamine, topical hydrocortisone, and all statins. All negative associations were replicated: loratadine HR 0.84 (95%CI 0.60-1.20), topical hydrocortisone HR 1.37 (95%CI 0.92-2.05), and statins HR 1.06 (95%CI 0.92-1.22). A positive association was not found between NSAIDS and hyperkalemia (HR 0.93 (95%CI 0.81-1.06)). The above positive and negative controls are reassuring for the ability of this claims database to detect clinically relevant hyperkalemia and to find a null result when no association is known.

The bolded warning for drospirenone and hyperkalemia also has the potential to introduce a measurement bias. This warning makes potassium monitoring in the drospirenone group more likely, inducing a bias away from the null. This anti-conservative bias provides additional confidence in our null result. If channeling bias is present in our study population, steering patients at high risk for hyperkalemia away from drospirenone would provide a bias toward the null. Another interpretation of the study results is that, based on the regulatory framework, current clinical practice is sufficient to mitigate the risk of hyperkalemia in this population.

Due to the nature of a claims database, residual confounding is always present. Alcohol consumption, ethnicity, and diet are all potential unadjusted confounders in our study. Two covariates in the analysis, smoking and obesity, are reported only to justify treatment (such as bariatric surgery or smoking cessation therapy) and are not completely controlled.

## Future implications

Although a clinically significant increase in the diagnosis of hyperkalemia was not found in our analysis, a subclinical increase in serum potassium in this population cannot be ruled out. Increased utilization of spironolactone in patients taking drospirenone and poor compliance with the requirement for potassium monitoring in the package insert suggests a lack of attention to the possibility of hyperkalemia. Spironolactone however has a strong association with hyperkalemia, and, for patients taking both spironolactone and drospirenone, it is concerning that so few physicians follow the package insert monitoring recommendations. This suggests that package inserts may not be an effective mechanism for the communication of drug safety information. If an increase in hyperkalemia had been found in patients taking drospirenone, this current monitoring practice may not have been sufficient to detect it.

## Conclusions

In a large cohort of young women, drospirenone did not cause a clinically significant increase in risk for hyperkalemia when compared with other progestin-containing OCs. It is however concerning that, despite the bolded warning for hyperkalemia, drospirenone and spironolactone are used together preferentially. This likely demonstrates a channeling of patients with premenstrual dysphoric disorder and polycystic ovary syndrome to use of drospirenone. Furthermore, physicians are not following the monitoring requirements for serum potassium assays in the package insert for patients taking these two medications.

## Author detail

STB and SRP work at the Center for Drug Evaluation and Research in the Food and Drug Administration. JMB is a professor of medicine and epidemiology at McGill University; ME is an assistant professor of medicine in the Pharmaceutical Outcomes Programme at the University of British Columbia; and JACD is an assistant professor of Pharmaceutical Outcomes & Policy at the University of Florida. XL is a graduate student in the department of Pharmaceutical Outcomes & Policy at the University of Florida.

## Competing interests

This manuscript represents the opinions of the authors and not those of the Food and Drug Administration. JMB is a physician scientist who receives peer review financial support from le Fonds de la Recherche en Santé du Québec. The authors have no other competing interests.

## Authors' contributions

JMB, JACD, and ME acquired data for this analysis. All authors contributed to the study design. The statistical analysis was completed by STB and JACD, and all authors contributed to the interpretation of the data. STB wrote the draft manuscript and all authors contributed to critical revision of the manuscript for important intellectual content. JACD is the study guarantor. All authors read and approved the final manuscript for publication.

## Pre-publication history

The pre-publication history for this paper can be accessed here:

http://www.biomedcentral.com/1472-6904/11/23/prepub
